# A Prediction Model for the Differential Diagnosis of Bowen's Disease and Bowenoid Actinic Keratosis

**DOI:** 10.1111/1346-8138.70450

**Published:** 2026-08-11

**Authors:** Byeonghyun Joo, Hyungrye Noh, Jin Ho Kim, Jong Hee Lee, Dongyoun Lee, Jun Young Kim, Jihye Park

**Affiliations:** ^1^ Department of Dermatology Samsung Medical Center, Sungkyunkwan University School of Medicine Seoul Republic of Korea; ^2^ Department of Dermatology Kyungpook National University Hospital, School of Medicine, Kyungpook National University Daegu Korea; ^3^ Department of Medical Device Management & Research, Samsung Advanced Institute for Health Sciences & Technology Sungkyunkwan University Seoul Republic of Korea

**Keywords:** actinic keratosis, Bowen's disease, diagnosis, differential, keratinocytes, prediction model

## Abstract

Bowen's disease and bowenoid actinic keratosis share overlapping clinical and histopathological features, and there is no clear consensus regarding their differential diagnosis. This study aimed to identify clinicopathological differences between the two diseases and to develop a diagnostic prediction model in a Korean population. We retrospectively analyzed 253 patients pathologically diagnosed with Bowen's disease or bowenoid actinic keratosis at a single institution in South Korea. Clinical and histopathological variables were compared between the two groups, and significant variables were incorporated into a multivariable logistic regression model. The model was externally validated using an independent cohort of 207 patients from another institution in South Korea. In the development cohort, variables significantly associated with diagnosis included lesion location, adjacent pigmented lesions, solar elastosis, pathologically identical lesions in the same region, and the number of atypical cells within a 3 mm width of the epidermis. The prediction model showed good discriminative performance, with an area under the receiver operating characteristic curve of 0.897. In the external validation cohort, the model demonstrated excellent performance, with an area under the curve of 0.994 and an accuracy of 95.17%. These findings suggest that integration of clinical and histopathological features may help differentiate Bowen's disease from bowenoid actinic keratosis in Korean clinical settings. Further validation in ethnically and geographically diverse populations is needed before broader application of the proposed model.

## 1 Introduction

Actinic keratosis (AK) is the most common precursor of cutaneous squamous cell carcinoma (SCC). Approximately 5%–10% of AK lesions have been reported to progress to SCC [1]. These lesions generally appear as multiple scaly erythematous papules or plaques on the skin that has been damaged by chronic ultraviolet (UV) ray exposure. They are usually smaller than 1 cm in diameter. Histologically, AK is characterized by focal parakeratosis, mild acanthosis with occasional downward budding, and cytologic atypia of the basal keratinocytes. Cytologic atypia can extend to the mid‐ to upper epidermis or along adnexal structures. It is usually accompanied by solar elastosis and mild inflammatory infiltration in the dermis [2]. AK lesions are further classified into several subtypes based on additional histological characteristics, including hypertrophic, atrophic, lichenoid, acantholytic, pigmented, proliferative, bowenoid, and pagetoid.

Bowen's disease (BD), first described in 1912, is defined as SCC in situ of the epidermis [3]. Clinically, BD presents as a slightly raised, large scaly erythematous plaque; it may resemble AK but, unlike AK, BD can also appear on areas of the skin not exposed to sunlight, such as the trunk and thigh [1]. BD usually manifests as a single lesion, although reports indicate that about 20% of BD cases may exhibit multiple lesions [1]. Histopathological features of BD include full‐thickness atypia of the epidermis, mitotic figures, and disorderly epidermis due to abnormal differentiation of epidermal keratinocytes [4, 5].

Among the subtypes of AK, bowenoid AK (bAK) shares similar histopathological characteristics with BD, exhibiting nearly full‐thickness atypia in the epidermis. Currently, major histological discriminators between the two diseases reveal that keratinocytic atypia in BD involves the follicular structure, whereas bAK is characterized by prominent solar elastosis in the dermis. However, since these features can overlap in both diseases and only partial characteristics of the lesion may be visible on skin biopsy, they are generally not utilized for differential diagnosis. Consequently, a clear consensus for distinguishing the two diseases is still needed.

Dermatopathologists assert that criteria for differential diagnosis between clinicians and pathologists are essential to provide appropriate treatment to patients by establishing specific terminologies to distinguish AK, BD, and SCC [6, 7]. Efforts to establish these criteria are ongoing, but consensus is often lacking on whether bAK should be equated with BD, if bAK is more likely to progress to SCC compared to other AKs, and if bAK should be treated more aggressively [8].

The diagnosis of skin cancer follows a specific process: a skin biopsy is performed when cancer is clinically suspected, and the tissue is subsequently examined by a pathologist. While some countries have dermatopathologists familiar with clinical practice, in most countries, dermatologists perform skin biopsy based on clinical suspicions and determine treatment based on the pathologist's diagnosis. Sometimes, a definitive diagnosis cannot be based solely on pathological features; in such instances, clinical features may be beneficial, or a consensus among pathologists may be needed. Nevertheless, challenges such as information exchange between departments and limited resources can complicate achieving sufficient consensus among pathologists and dermatologists.

Especially in cases like bAK and BD, which exhibit many clinical and pathological similarities, reliable diagnostic methods are essential. The distinctions between them stem from varying degrees of histological atypia and clinical features, rendering differentiation based solely on pathological or clinical observations challenging. Consequently, collaborative diagnosis and information sharing between clinical and pathological departments are crucial.

For these reasons, we aimed to develop a predictive model that distinguishes between the two diseases by integrating various clinical and pathological factors. Our objective is to enhance diagnostic accuracy and facilitate appropriate treatment based on the diagnosis.

## 2 Methods

### 2.1 Study Population for Model Development

A retrospective cohort of patients diagnosed pathologically with BD or bAK at a single center (Samsung Medical Center in Seoul, South Korea) between June 2018 and June 2023 was included in the development group. We obtained approval from the Institutional Review Board of Samsung Medical Center (2023‐06‐027). No written consent has been obtained from the patients as there is no patient‐identifiable data included.

### 2.2 Data Collection

Patient demographic data and clinical and histopathological features of their skin lesions were collected from electronic medical records. Demographic variables included age and sex. Clinical features encompassed size, location, presence of scale, border regularity, multiplicity of other cutaneous neoplasms, and the presence of adjacent pigmented or erythematous spots. Histologically, we examined the number of atypical cells and mitotic figures within a 3 mm‐width of the epidermis (since the skin biopsy was conducted with a punch tool at least 3 mm wide), thickness of the stratum corneum, degree of solar elastosis, degree of loss of polarity, capillary dilatation, degree of parakeratosis, degree of lymphocytic infiltrates, and the pattern of inflammatory cell infiltrates. Histopathological variables were retrospectively assessed using hematoxylin and eosin‐stained biopsy slides according to predefined criteria. The same variable definitions and prediction formula derived from the development cohort were applied to the external validation cohort.

### 2.3 Definition of Study Variables

Demographic, clinical, and histopathological data were further categorized into several subgroups according to our predefined criteria (Table 1). For solar elastosis, we used a simplified three‐tier classification (none, partial, and marked) based on the extent of dermal involvement to improve reproducibility in routine assessment.

**TABLE 1:** Definition of study variables.

Study variables	Definition
Location	
Non sun‐exposed area	The body part that is typically covered by clothing, including external genitalia, groin, buttock, and thigh
Intermittent sun‐exposed area	The body part where the degree of sun exposure varies with the season, including forearm, neck, and lower leg
Chronic sun‐exposed area	The body part consistently exposed to sunlight, including the face and scalp
Adjacent lesions	
Pigmented spots	Whether pigmented spots are observed around the original lesion in clinical photos, potentially including lentigines, freckles, melasma, and pigmented actinic keratosis
Erythematous lesions	Whether erythematous papules and patches are observed around the original lesion in clinical photos
Both pigmented spots and erythematous lesions	Whether pigmented spots and erythematous lesions appear around the original lesion in clinical photos
Multiplicity of other skin lesions	The presence of other cutaneous neoplasms in the same or different anatomical regions, pathologically confirmed
Same lesion	The presence of a pathologically identical lesion. In cases of BD, same lesions refer to BD; in cases of bAK, same lesions refer to bAK.
Counterpart	The presence of a counterpart to the original lesion. In the case of BD, the counterpart lesion is bAK and vice versa
Nonmelanoma skin cancer	The presence of non‐melanoma skin cancer, including BCC and SCC
Melanoma	The presence of pathologically proven melanoma
Atypical cell within a 3 mm‐width of the epidermis	The number of atypical cells observed within a 3 mm‐width of the epidermis, including dysmorphic and necrotic keratinocytes
Atypical mitosis within a 3 mm‐width of the epidermis	The number of mitotic figures observed within a 3 mm‐width of the epidermis
Solar elastosis	
None	The case when basophilic degeneration of dermal collagen fibers is not observed
Partial	Biopsy slides show spatially < 50% basophilic degeneration of collagen fibers in the dermis
Marked	Biopsy slides show spatially > 50% basophilic degeneration of collagen fibers in the dermis
Loss of polarity	
None	The condition where there is no loss of polarity among basal keratinocytes
Partial	Biopsy slides show loss of polarity in < 50% of basal keratinocytes
Marked	Biopsy slides show loss of polarity in > 50% of basal keratinocytes
Parakeratosis	
None	The condition where parakeratotic changes in the stratum corneum are not observed
Partial	Biopsy slides show spatially < 50% parakeratotic changes in the stratum corneum
Marked	Biopsy slides show spatially > 50% parakeratotic changes in the stratum corneum
The degree of lymphocytic infiltrate	
None	Cases where dermal lymphocytic infiltrate is not observed
Partial	Spatial occupation of less than 80% by lymphocytes in the papillary dermis observed on biopsy slides
Marked	Spatial occupation of more than 80% by lymphocytes in the papillary dermis observed on biopsy slides

Abbreviations: bAK, bowenoid actinic keratosis; BCC, basal cell carcinoma; BD, Bowen's disease; SCC, squamous cell carcinoma.

### 2.4 Statistical Analysis

Statistical analysis was performed to identify significant differences between the two diseases, employing univariate analysis. The Student's *t*‐test was used for numerical data, while the chi‐squared test was used to compare categorical data between the two disease groups. Variables demonstrating significant differences (*p* < 0.05) were selected for further multivariate analysis.

The relationship among multiple variables was established using logistic regression analysis, and the corresponding coefficients were calculated. A differential diagnostic prediction model was then developed using selected variables, with coefficients derived from multiple logistic regression analysis. Internal validation was performed using 10‐fold cross‐validation. To determine the optimal cutoff value, predicted probabilities were calculated from the logistic regression model, and the cutoff was selected using the closest top‐left method on the receiver operating characteristic curve. Subsequently, the area under the curve (AUC) and 95% confidence intervals (CIs) were determined. The external cohort comprised patients who were pathologically diagnosed with BD or bAK during the same period (June 2018 to June 2023) at another institution.

All statistical analyses were conducted using R Statistical Software (version 3.6.3; Foundation for Statistical Computing, Vienna, Austria). Statistical significance for all analyses was established at *p* < 0.05.

## 3 Results

### 3.1 Statistical Analysis of Multiple Variables in the Development Group

At a single center, 330 patients pathologically diagnosed with BD or bAK were initially considered for the cohort. However, 77 patients were excluded: 32 due to unavailable biopsy slides, 21 lacking clinical photos of their lesions, 14 for having low‐quality biopsy slides, and 10 for inadequate clinical data (Figure 1).

**FIGURE 1:**
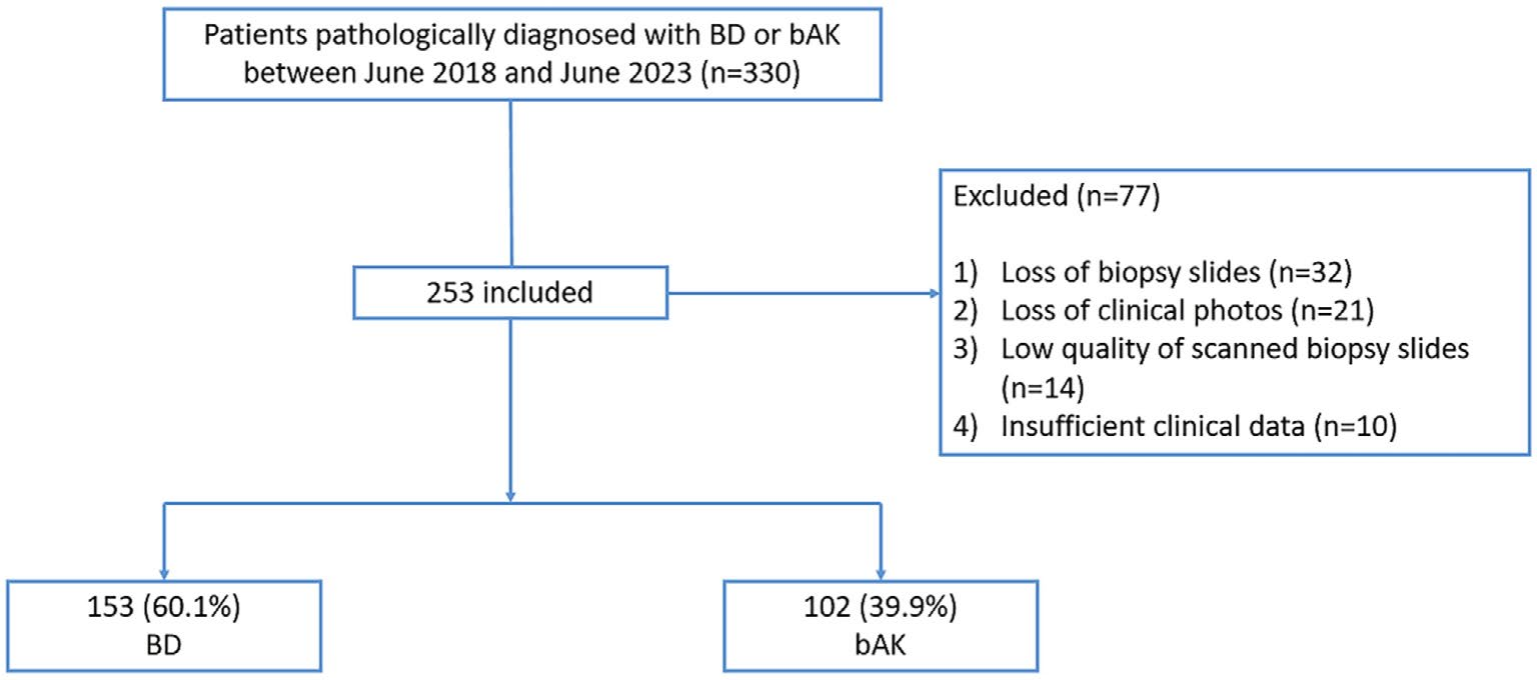
Flowchart of cohort selection. bAK, bowenoid actinic keratosis; BD, Bowen's disease.

Consequently, 152 BD patients and 101 bAK patients were included in the development group. Univariate analysis revealed no significant differences in lesion size, scale presence, stratum corneum thickness, loss of polarity, lymphocytic infiltrate patterns, parakeratosis degree, and capillary dilatation between the BD and bAK groups.

Conversely, the percentage of females was notably higher in the bAK group than in the BD group (68.3% vs. 45.4%, *p* = 0.001), and the patients in the bAK group were older on average than those in the BD group (79.04 years vs. 70.36 years, *p* < 0.001) (Table 2).

**TABLE 2:** Univariate analysis of the development group (*n* = 253).

	Bowen's disease (BD) (*n* = 152)	Bowenoid actinic keratosis (bAK) (*n* = 101)	*p*
Age, years, mean (range)	70.36 (28–94)	79.04 (49–96)	**< 0.001**
Sex, *n* (%)			
Male	83 (54.6)	32 (31.7)	**0.001**
Female	69 (45.4)	69 (68.3)	
Size, (cm), mean (SD)	1.22 (0.91)	1.08 (0.65)	0.187
Scale, *n* (%)	70 (46.1)	43 (42.6)	0.677
Irregular border, *n* (%)	60 (39.5)	54 (53.5)	**0.039**
Location, *n* (%)			**< 0.001**
Non sun‐exposed area	59 (38.8)	2 (2.0)	
Intermittently sun‐exposed area	38 (25.0)	8 (7.9)	
Chronically sun‐exposed area	55 (36.2)	91 (90.1)	
Adjacent lesions, *n* (%)			**< 0.001**
Pigmented spots	25 (16.4)	41 (40.6)	
Erythematous lesions	16 (11.8)	15 (14.9)	
Both pigmented and erythematous spots	9 (5.9)	22 (21.8)	
Multiplicity of cutaneous neoplasms, *n* (%)	52 (34.2)	57 (56.4)	**< 0.001**
Same anatomical region, *n* (%)			
Identical lesion	10 (6.6)	23 (22.8)	**< 0.001**
Counterpart	2 (1.3)	4 (4.0)	0.351
Nonmelanoma skin cancer	0 (0.0)	2 (2.0)	0.309
Melanoma	0 (0.0)	0 (0.0)	NA
Other anatomical regions, *n* (%)			
Identical lesion	23 (15.1)	30 (29.7)	**0.008**
Counterpart	17 (11.2)	8 (7.9)	0.524
Nonmelanoma skin cancer	17 (11.2)	11 (10.9)	1
Melanoma	1 (0.7)	0 (0.0)	1
Atypical cells within a 3 mm‐width of the epidermis, mean (SD)	13.68 (20.05)	5.51 (7.06)	**< 0.001**
Atypical mitoses within a 3 mm‐width of the epidermis, mean (SD)	7.22 (15.63)	2.10 (3.63)	**0.001**
Thickness of the stratum corneum, mean (mm) (SD)	0.23 (0.39)	0.28 (0.44)	0.35
Solar elastosis, *n* (%)			**< 0.001**
None	101 (66.4)	6 (5.9)	
Partial	32 (21.1)	44 (43.6)	
Marked	19 (12.5)	51 (50.5)	
Loss of polarity, *n* (%)			0.148
None	109 (71.7)	82 (81.2)	
Partial	37 (24.3)	18 (17.8)	
Marked	6 (3.9)	1 (1.0)	
Lymphocytic infiltrate, *n* (%)			**0.044**
None	4 (2.6)	4 (4.0)	
Partial	113 (74.3)	60 (59.4)	
Marked	35 (23.0)	37 (36.6)	
Pattern of lymphocytic infiltrates, *n* (%)			0.46
None	3 (2.0)	4 (4.0)	
Scanty	22 (14.5)	21 (20.8)	
Lichenoid	46 (30.3)	23 (22.8)	
Perivascular	73 (48.0)	47 (46.5)	
Mixed	8 (5.3)	6 (5.9)	
Parakeratosis, *n* (%)			0.368
None	33 (21.7)	24 (23.8)	
Partial	78 (51.3)	43 (42.6)	
Marked	41 (27.0)	34 (33.7)	
Capillary dilation, *n* (%)	75 (49.3)	42 (41.6)	0.279

*Note:* Bold values indicate statistical significance (*p* < 0.05).

Abbreviation: SD, standard deviation.

In terms of clinical data, the incidence of lesions with irregular borders was higher in the bAK group (53.5% vs. 39.5%, *p* = 0.039), and lesions were more frequently located in sun‐exposed areas in the bAK group (*p* < 0.001). Additionally, the prevalence of lesions with adjacent pigmented spots or erythematous skin was higher in the bAK group (*p* < 0.001), along with a greater incidence of multiple cutaneous neoplasms (56.4% vs. 34.2%, *p* < 0.001) (Table 2).

Histologically, the number of atypical cells within a 3 mm‐width of the epidermis was higher in the BD group (13.68 vs. 5.51, *p* < 0.001), and the number of mitotic figures was similarly higher in the BD group (7.22 vs. 2.10, *p* = 0.001). However, the extent of solar elastosis was greater in the bAK group (*p* < 0.001), as was the degree of lymphocytic infiltrates (*p* = 0.044) (Table 2).

Several variables were significant in univariate analysis; therefore, multivariable logistic regression was performed to identify independent factors associated with bAK. Statistically significant factors included the anatomical location of the lesion, presence of adjacent pigmented or erythematous skin lesions, degree of solar elastosis, presence of pathologically identical skin tumors in the same region, and the number of atypical cells within a 3 mm‐width of the epidermis (*p* < 0.05).

Additionally, the odds ratios (OR) and coefficients for each variable were calculated, with higher values indicating a greater probability of bAK. Among the categorical variables, the most discriminative categorical variables was solar elastosis involving more than 50% of the dermal area (OR, 12.61; 95% CI, 4.05–39.30; *p* < 0.0001), followed by chronic sun exposure (OR, 9.18; 95% CI, 1.76–47.95; *p* = 0.01), solar elastosis involving less than 50% of the dermal area (OR, 9.09; 95% CI, 3.13–26.39; *p* < 0.0001), identical disease in the same region (OR, 3.94; 95% CI, 1.23–12.61; *p* = 0.02), pigmented spots in adjacent areas (OR, 2.80; 95% CI, 1.16–6.78; *p* = 0.02) (Table 3). Additionally, the estimated OR and 95% CI for the number of atypical cells within a 3 mm‐width of the epidermis, a continuous variable, were 0.95 and 0.91–0.99, respectively, with a *p*‐value of 0.01. Unlike the other variables, a greater number of atypical cells within a 3 mm‐width of the epidermis was associated with a higher likelihood of BD, because the model outcome was bAK and an OR below 1 indicates lower odds of bAK.

**TABLE 3:** Multivariable logistic regression model for differentiating Bowenoid actinic keratosis from Bowen's disease.

Variable	Odds ratio	95% CI	*p*
Intermittent sun‐exposed area	3.17	0.51–19.58	0.21
Chronic sun‐exposed area	9.18	1.76–47.95	0.01
Solar elastosis, partial	9.09	3.13–26.39	< 0.0001
Solar elastosis, marked	12.61	4.05–39.30	< 0.0001
Pathologically identical lesion in the same region	3.94	1.23–12.61	0.02
Adjacent pigmented spots	2.80	1.16–6.78	0.02
Adjacent erythematous lesions	1.49	0.51–4.34	0.47
Both pigmented and erythematous lesions	1.87	0.65–5.39	0.25
Number of atypical cells	0.95	0.91–0.99	0.01

*Note:* Odds ratios > 1 indicate a higher likelihood of Bowenoid actinic keratosis, whereas odds ratios < 1 indicate a higher likelihood of Bowen's disease.

Abbreviation: CI, confidence interval.

### 3.2 Establishment of a Differential Diagnostic Model

Variables extracted from multiple logistic regression analysis can be largely categorized as follows: the location of the lesion, the existence of adjacent lesions, the degree of solar elastosis, whether there is a pathologically identical lesion near the original lesion, and the number of atypical cells within a 3 mm‐width of the epidermis. Based on these, a differential diagnostic model was developed. Overall, a complete formula for the estimated model was constructed using coefficients from multiple logistic regression analysis. This multivariate prediction model was illustrated with a nomogram (Figure S1).

For internal validation, 10‐fold cross‐validation was performed, and the mean ROC curve was generated. The AUC was 0.897 (95% CI = 0.859–0.934) (Figure 2). To determine the optimal cutoff value, predicted probabilities were calculated using the logistic regression model, and the cutoff was selected using the closest top‐left method. The optimal cutoff value was 0.5, yielding a sensitivity of 90% and a specificity of 81%. Thus, according to this formula, lesions with a predicted probability of bAK greater than 0.5 were classified as bAK, whereas those with a value below 0.5 were classified as BD.

**FIGURE 2:**
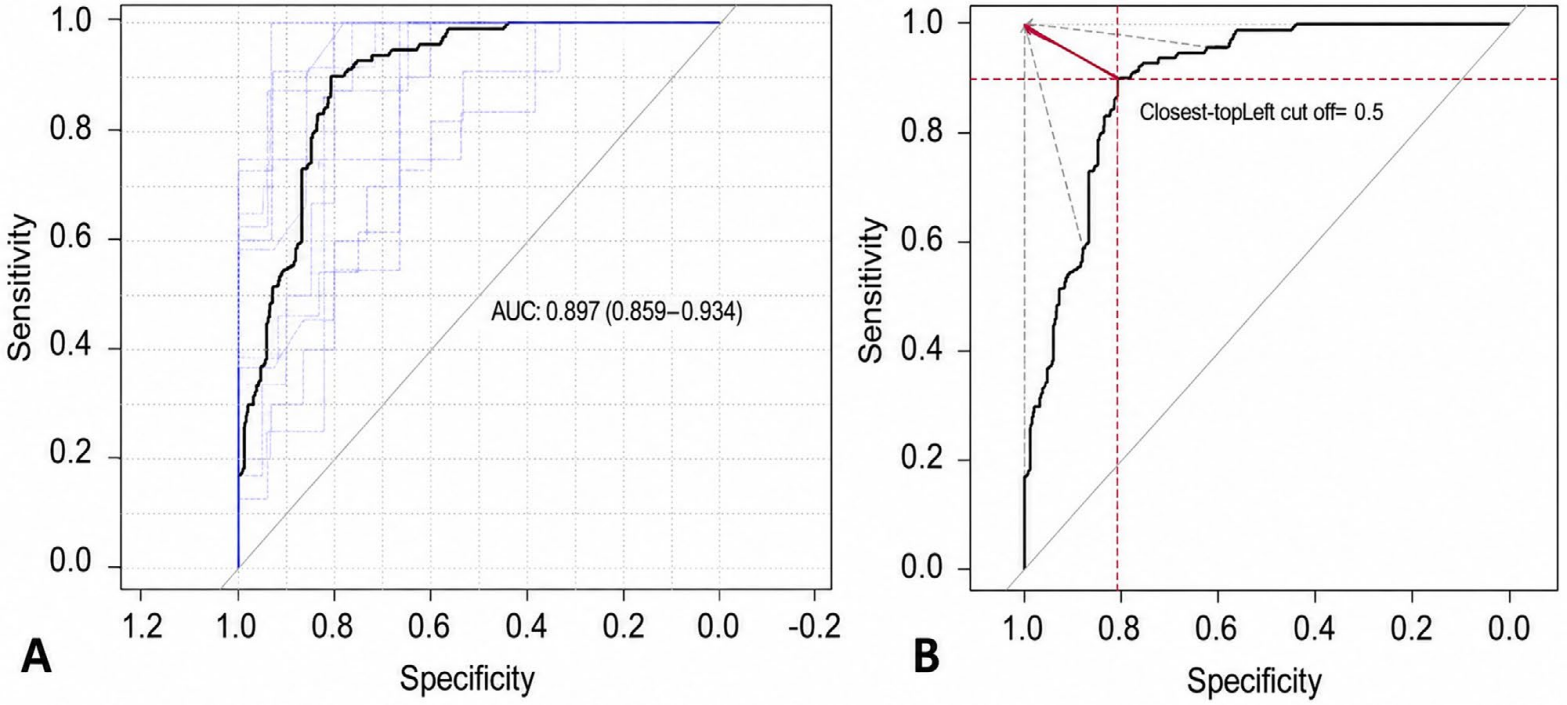
Evaluation of the prediction model. (A) K‐fold (*K* = 10) cross‐validation of model. After completing 10 cycles of cross‐validation, the average sensitivity and specificity were estimated and are represented by a black line. (B) The ideal cutoff value was calculated to be 0.5 using the closest top‐left method. AUC, area under the curve.

### 3.3 External Validation of the Developed Prediction Model

For external validation of our novel prediction model, we applied our model to data from another institution. Based on an expected AUC range of 0.85–0.95, we determined that a minimum of 188 patients from other centers was required for external validation. Consequently, we enrolled an additional 207 cases (169 BD cases and 38 bAK cases) diagnosed from June 2018 to 2023 at another center, Kyungpook National University Hospital in Daegu, South Korea.

Using external data, ROC analysis was conducted to estimate the discriminant power of our model. The ROC curve was subsequently drawn, and an AUC value of 0.9941 (95% CI = 0.9873–1) was calculated (Figure 3). Using an internally derived cutoff value, the ROC curve demonstrated a sensitivity of 97.4% and specificity of 94.7% (Figure 3). The model's accuracy was computed at 95.17% (95% CI, 91.3–97.66). These findings indicate that the prediction model provides a high level of discrimination between the two diseases.

**FIGURE 3:**
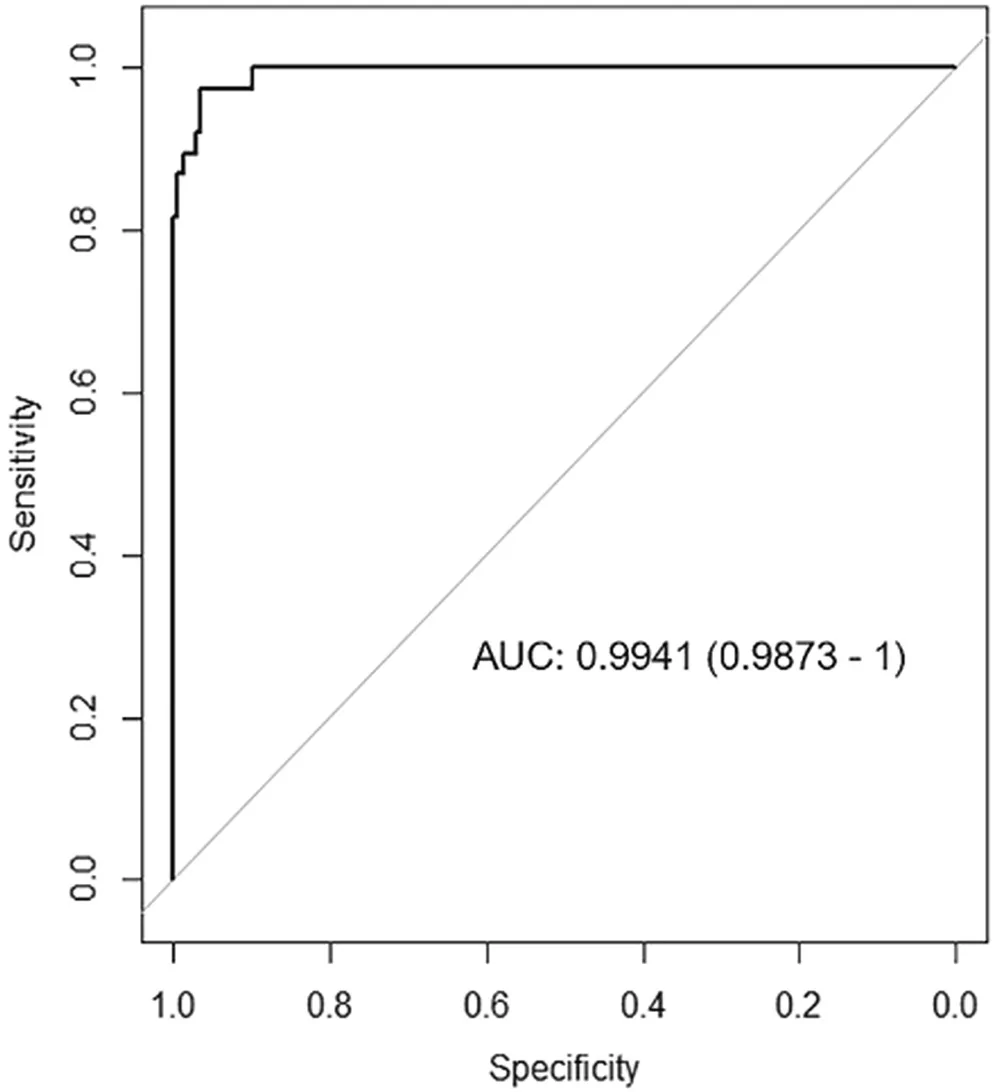
External validation of the prediction model. The receiver operating characteristic (ROC) curve was plotted. AUC, area under the curve.

## 4 Discussion

Although BD and bAK share many clinical and histopathological features, their therapeutic implications differ. In the case of BD, which is considered a carcinoma, surgical interventions such as wide excision and Mohs micrographic surgery (MMS) are prioritized. Conversely, bAK, being a precancerous lesion, is predominantly treated with nonsurgical approaches including curettage, cryotherapy, and topical agents such as 5‐fluorouracil and diclofenac [9, 10, 11].

Therefore, accurate differential diagnosis of the two diseases is crucial, and several efforts have been made to distinguish them [12, 13, 14]. For instance, a previous study attempted to differentiate between the two diseases using immunohistochemistry (IHC) with markers p16 and p63. Although it successfully differentiated between common AK and SCC, BD and bAK exhibited similar IHC patterns [15].

The histological appearance of cutaneous neoplasms can vary based on the tissue segment obtained and the extent of sampling in actual clinical practice. Consequently, pathologists face challenges in accurately distinguishing these diseases based solely on histology. This has made their differential diagnosis a major focal point in discussions among dermatologists and dermatopathologists.

Ramos‐Ceballos FI, et al. introduced a three‐tiered keratinocytic intraepithelial neoplasia (KIN) diagnostic system based on the degree of cytologic atypia [16]. The KIN system categorizes the disease by histological progression, similar to the cervical intraepithelial neoplasia (CIN) system used in gynecology. Using the KIN system, multiple anatomic pathologists, dermatologists, and dermatopathologists reviewed 15 biopsy slides with mixed AK and BD cases, achieving high diagnostic concordance. However, the study had limitations, including a small sample size and a focus solely on pathological characteristics of each disease. Our study addressed these limitations by involving a total sample of 460 patients, including an external validation group, and developing a prediction model that incorporates clinical features. The results of this study provide insights into the medical information that should be shared between clinicians and pathologists. Additionally, since our study includes cases diagnosed as bAK, which are pathologically similar to BD and thus challenging to differentiate, our prediction model is expected to distinguish even the more ambiguous cases.

Despite the high accuracy of the predictive model developed in this study, several limitations remain. First, we used variables with broad meanings, such as “pigmented spots around the original lesion” and “erythematous lesion around the original lesion.” These encompassed a diverse group of findings, with the former including freckles, melasma, nevi, and pigmented AK, and the latter encompassing AK, lichenoid keratosis, folliculitis, and nonspecific erythematous spots. Second, this prediction model was developed using data exclusively from Asian patients. Therefore, the model should be considered population‐specific, which represents an important limitation of the present study. Notably, the prevalence of bAK is closely associated with UV exposure, which varies across ethnic groups [17, 18, 19]. Consequently, the applicability of our model to non‐Asian populations, particularly Caucasian populations with a high incidence of skin cancer, requires validation through international studies.

Despite these limitations, our study has several strengths. First, it identified statistically significant clinicopathological differences between BD and bAK. In bAK, clinical variables related to UV exposure and their corresponding histopathological features, such as solar elastosis, were more prominently observed. In contrast, patients with BD were younger than those with bAK, and BD showed more pronounced SCC‐like histopathologic features, including cytologic atypia and atypical mitoses. Second, we proposed a novel prediction model for distinguishing BD from bAK by integrating clinical and histopathological variables. Another strength of this study is the external validation of the model using an independent cohort from another institution. Although the very high AUC observed in the external validation supports the strong discriminative ability, this finding should be interpreted cautiously, as differences in cohort composition or a possible spectrum effect may have contributed to the result. Further validation in broader clinical settings, along with prospective studies, will be necessary to confirm the robustness and generalizability of the model.

In conclusion, our findings suggest that integrating multiple clinical and histopathological features may help differentiate BD from bAK with a high level of accuracy.

## Funding

The authors have nothing to report.

## Ethics Statement

The study was approved by the reviewed and approved by Samsung Medical Center IRB (approval #2023‐06‐027).

## Consent

The authors have nothing to report.

## Conflicts of Interest

The authors declare no conflicts of interest.

## Supporting information


**Figure S1:** The results of multivariate analysis illustrated as a nomogram. Using the value of each variable, the probability of bAK can be calculated from the total points. Conversely, the probability of BD is calculated as 1—(probability of bAK).

## Data Availability

The data that support the findings of this study are available from the corresponding author upon reasonable request.
